# Supplementary material for: Rapid recycling of glutamate transporters on the astroglial surface
Source: eLife. 2021 Apr 16;10:e64714. doi: 10.7554/eLife.64714 (PMC8079145; doi:10.7554/eLife.64714)
Supplement: Transparent reporting form [file elife-64714-transrepform.docx]

***eLife’s* transparent reporting form**

We encourage authors to provide detailed information *within their submission* to facilitate the interpretation and replication of experiments. Authors can upload supporting documentation to indicate the use of appropriate reporting guidelines for health-related research (see [EQUATOR Network](http://www.equator-network.org/%20)), life science research (see the [BioSharing Information Resource](https://biosharing.org/)), or the [ARRIVE guidelines](http://www.plosbiology.org/article/info:doi/10.1371/journal.pbio.1000412) for reporting work involving animal research. Where applicable, authors should refer to any relevant reporting standards documents in this form.

If you have any questions, please consult our Journal Policies and/or contact us: [editorial@elifesciences.org](mailto:editorial@elifesciences.org).

**Sample-size estimation**

- You should state whether an appropriate sample size was computed when the study was being designed
- You should state the statistical method of sample size computation and any required assumptions
- If no explicit power analysis was used, you should describe how you decided what sample (replicate) size (number) to use

Please outline where this information can be found within the submission (e.g., sections or figure legends), or explain why this information doesn’t apply to your submission:

The sample size was not predetermined because the variability of measured parameters, be it the distribution moments or the unexplained variance for non-linear curve fitting, was not known a priori, and there was no known restriction or bias in the cohort sampling. Sampling was carried out in accord with established conventions in cell imaging, ROI sampling procedures were detailed throughout (Figure legends, Materials and Methods).

**Replicates**

- You should report how often each experiment was performed
- You should include a definition of biological versus technical replication
- The data obtained should be provided and sufficient information should be provided to indicate the number of independent biological and/or technical replicates
- If you encountered any outliers, you should describe how these were handled
- Criteria for exclusion/inclusion of data should be clearly stated
- High-throughput sequence data should be uploaded before submission, with a private link for reviewers provided (these are available from both GEO and ArrayExpress)

Please outline where this information can be found within the submission (e.g., sections or figure legends), or explain why this information doesn’t apply to your submission:

No repeated measures were included in testing biological hypotheses. FPAR readout involved live imaging sessions generating a single analysis outcome per test. dSTORM followed established procedures for nanoscale analyses (EM, etc.) in fixed tissue, in which within-prep variance dwarfs between-prep variance. Sample sizes and statistical inference detail are specified in the text and figure legend throughout.

**Statistical reporting**

- Statistical analysis methods should be described and justified
- Raw data should be presented in figures whenever informative to do so (typically when N per group is less than 10)
- For each experiment, you should identify the statistical tests used, exact values of N, definitions of center, methods of multiple test correction, and dispersion and precision measures (e.g., mean, median, SD, SEM, confidence intervals; and, for the major substantive results, a measure of effect size (e.g., Pearson's r, Cohen's d)
- Report exact p-values wherever possible alongside the summary statistics and 95% confidence intervals. These should be reported for all key questions and not only when the p-value is less than 0.05.

Please outline where this information can be found within the submission (e.g., sections or figure legends), or explain why this information doesn’t apply to your submission:

Statistical methods are detailed throughout the text, including ROI sampling (Methods lines 882-894, 947-953), integral geometry estimates such as the clustering analysis (Methods lines 954-960), and a paired-sample protocol to suppress Bayesian-type bias (Fig. 4B-C). Statistical data are presented as mean ± SEM or mean ±95% confidence interval as specified.

(For large datasets, or papers with a very large number of statistical tests, you may upload a single table file with tests, Ns, etc., with reference to sections in the manuscript.)

**Group allocation**

- Indicate how samples were allocated into experimental groups (in the case of clinical studies, please specify allocation to treatment method); if randomization was used, please also state if restricted randomization was applied
- Indicate if masking was used during group allocation, data collection and/or data analysis

Please outline where this information can be found within the submission (e.g., sections or figure legends), or explain why this information doesn’t apply to your submission:

The work involved no cohort or clinical studies. Sampling methods were designed to avoid observational bias and are detailed throughout the text and figures, as described above.

**Additional data files (“source data”)**

- We encourage you to upload relevant additional data files, such as numerical data that are represented as a graph in a figure, or as a summary table
- Where provided, these should be in the most useful format, and they can be uploaded as “Source data” files linked to a main figure or table
- Include model definition files including the full list of parameters used
- Include code used for data analysis (e.g., R, MatLab)
- Avoid stating that data files are “available upon request”

Please indicate the figures or tables for which source data files have been provided:

The additional Excel data files "Figure X_and_Supplement_ Source.xlsx" contains original numerical data embedded in the main figure X and Supplements, in the corresponding figure panels.
